# Determinants of advance-directive completion for end-of-life preparedness among older Korean adults

**DOI:** 10.3389/fpubh.2026.1839072

**Published:** 2026-06-30

**Authors:** Hyun-Sook Lee, Sang-Mi Kim

**Affiliations:** 1 Department of Health Administration, Kongju National University, Gongju, Republic of Korea; 2 Department of AI Health Information Management, Yonsei University, Wonju, Republic of Korea

**Keywords:** advance care planning, advance-directive completion, end-of-life preparedness, older Korean adults, South Korea

## Abstract

**Aim:**

This study analyzed determinants of advance-directive (AD) completion for end-of-life preparedness among older Korean adults.

**Methods:**

The study used data from 9,796 individuals in the 2023 National Survey of Older Koreans. Complex-sample weighted analyses and multivariate logistic regression were conducted to identify factors associated with AD completion among older Koreans. Sociodemographic, health-status, and death-preparation characteristics were analyzed.

**Results:**

Multivariate analysis revealed that the strongest predictors of AD completion were organ- or tissue-donation willingness, educational attainment, and writing a will. Other significant factors included funeral consultation and death-preparation education.

**Conclusion:**

These findings suggest that death-preparation behaviors and end-of-life planning activities are significantly associated with AD completion among older Korean adults. Public-health strategies should support targeted education and communication interventions to improve older adults’ engagement in advanced care planning.

## 1 Introduction

According to Statistics Korea, life expectancy for men and women increased from 58.7 and 65.8 years in 1970 to 79.9 and 85.6 years in 2022, respectively (1). Korea is projected to rank among the countries with the highest life expectancy by 2030 (2). Since the 1970s, many countries have institutionalized measures to ensure human dignity and a peaceful death. In Korea, the “Act on Hospice and Palliative Care and Decisions on Life-Sustaining Treatment for Dying Patients” was enacted in 2016 and came into effect in 2018 (3). Decision-making regarding life-sustaining treatment is a core bioethical issue centered on respect for patient autonomy. Korea’s Life-Sustaining Treatment Decision System allows individuals to withhold or withdraw futile life-prolonging interventions; public awareness regarding this system increased from 74.2% in 2019 to 90.1% in 2022 (4). A key initial step in this process is the completion and registration of advance directives (ADs) (5). The number of institutions that register ADs increased from 291 in 2018 to 686 in 2023 (6), indicating active end-of-life preparation among older Koreans.

Previously, completion of ADs was regarded as a key step for safeguarding patient autonomy in end-of-life decision-making. However, concerns have been raised regarding the limitations of relying solely on ADs as standard legal forms often fail to capture patients’ specific preferences and decision-making needs at the actual end-of-life stage (7–10). To address these limitations, the paradigm has shifted toward advanced care planning (ACP)—an ongoing, communicative process among patients, families, and healthcare professionals (11). As Fried et al. have demonstrated, ACP should be understood not as a single discrete event but as a dynamic process of health-behavior change (12). Within this contemporary framework, grounded in the theory of planned behavior (TPB) (13), AD completion may be understood not merely as an isolated administrative task but as an initial behavioral indicator of engagement in the ACP process (14, 15). Particularly among older adults, completing ADs is closely associated with a range of behavioral and cultural factors—including preparedness for death, end-of-life values, funeral decision-making, and organ-donation intentions (16). While recent Korean studies have explored AD-completion factors (17–21), a critical gap remains in the literature regarding how multifaceted, practical death-preparation behaviors directly determine AD completion among community-dwelling older adults. Most existing research has predominantly focused on clinical populations or has treated AD completion in a vacuum, limited to identifying simple correlations with sociodemographic variables rather than examining behavioral integration. Consequently, research is lacking on the behavioral determinants of AD completion from a holistic “end-of-life preparedness” perspective.

Critically, end-of-life preparedness encompasses both clinical/legal decisions (e.g., ADs) and social/ritual behaviors (e.g., funeral preparations, writing a will, selecting a burial site). In the East Asian and Korean cultural context, these actions are closely interconnected. The concept of a “good death” for older Koreans is deeply rooted in both exerting medical autonomy to minimize physical suffering and fulfilling a familial duty to reduce the emotional and socioeconomic burden on their children. Therefore, to better understand factors associated with AD completion, it is essential to simultaneously analyze how social and ritual death-preparation behaviors interact with clinical directives.

This study used the 2023 National Survey of Older Koreans (22) to identify AD-completion factors among older Koreans. Specifically, it examines how sociodemographic, health, and death-preparation characteristics affect AD completion for end-of-life readiness. The study extends previous research examining ADs in isolation and highlights the significance of AD completion within the broader context of ACP.

Through this analysis, we seek to provide foundational empirical evidence to support the future institutionalization of ACP and the development of community-based educational programs that promote autonomy in end-of-life decision-making.

## 2 Materials and methods

### 2.1 Study design

This is a cross-sectional analysis using complex-sample data to identify factors in the completion of ADs among older Koreans.

### 2.2 Data collection and participants

This study utilized cross-sectional data from the 2023 National Survey of Older Koreans, a follow-up to the National Survey on the Living Conditions and Welfare Needs of Older Adults initiated in 1994. Legally mandated under Article 5 of the Elderly Welfare Act, the survey is conducted every three years to inform policies on welfare and demographic transitions among older people. The 2023 survey applied stratified cluster sampling with primary probability proportional sampling and secondary systematic sampling across 17 provinces. It maintained continuity with prior surveys while revising items to reflect changing living conditions and removing outdated questions (22).

From a total of 10,078 respondents in the 2023 survey, this study analyzed data from 9,796 individuals after excluding cases with dementia diagnoses (155 respondents) and missing data (127 respondents) related to the completion of ADs and other study variables.

### 2.3 Ethical considerations

The data used in this study were publicly available for research purposes and de-identified to ensure anonymity. Ethical review exemption was granted by the Institutional Review Board (IRB) of Kongju National University (KNU_IRB_2025–034). Data were downloaded following the procedures of the Korea Institute for Health and Social Affairs after obtaining consent for information utilization, and analyses were conducted according to the provided guidelines.

### 2.4 Measurements

#### 2.4.1 Advance directive completion

The dependent variable in this study was the completion of ADs. Respondents answered “yes” or “no” to the question, “Have you prepared or are you preparing an advance directive to prepare for the end-of-life stage?”

#### 2.4.2 Sociodemographic characteristics

Sociodemographic characteristics examined in this study encompassed gender (male and female), age categorized into four groups (65–69 years, 70–74 years, 75–79 years, and 80 years or older), and educational level classified into five levels (no formal education, elementary school graduate, middle school graduate, high school graduate, and college or higher). Household size was determined based on the number of people living together continuously for at least 3 months (1–2 persons, 3 persons, or 4 or more persons). Religion was classified into either having no religion or having a religious affiliation.

Satisfaction was measured using five questions that assessed personal health status, financial status, social, leisure, and cultural activities, social relationships (such as interactions with neighbors and friends), and overall life satisfaction. Each of these dimensions was assessed using a 5-point Likert scale ranging from 1 (very dissatisfied) to 5 (very satisfied). Higher scores indicate higher satisfaction.

The burden of medical and caregiving expenses was determined by respondents’ indication of such expenses as the most significant financial burden among household expenditures. Specifically, “medical and care-related expenses” excluded health insurance premiums. “Nursing and caregiving expenses” included healthcare expenses, long-term care insurance out-of-pocket payments, personal caregiving expenses, and related supplies. Respondents who indicated such expenses as burdensome were classified as experiencing a burden.

The payer of medical expenses over the past year was categorized into four distinct groups: no medical expenses incurred, self or spouse (covering all or most expenses, with occasional assistance from children in special circumstances), children (covering either some portion or all of the medical expenses), and other payers (primarily supported by relatives other than children or social welfare institutions).

#### 2.4.3 Health-status characteristics

Health status characteristics assessed consisted of subjective health status, number of chronic diseases, number of prescribed medications, depression level, suicidal ideation, and history of falls.

Subjective health status was measured on a 5-point Likert scale, with higher scores indicating better health status. The number of chronic diseases included conditions diagnosed by a doctor that persist for 3 months or longer. In total, there were 33 chronic conditions excluding dementia. The number of chronic diseases was categorized as 0, 1, 2, or 3 or more.

The number of prescribed medications was determined by the number of medications prescribed by a physician and taken consistently for 3 months or longer, categorized as none, one medication, two medications, or three or more medications. Depression level was evaluated using the Korean version of the Short Form Geriatric Depression Scale (SGDS-K), consisting of 15 items. Each positive response was assigned a score of 1, and each negative response a score of 0, yielding a total score ranging from 0 to 15. Scores of 0–5 indicated no depression, 6–9 indicated moderate depressive symptoms, and scores of 10 or higher indicated severe depressive symptoms (23). Additionally, responses were recorded for suicidal ideation (yes or no) and history of falls (yes or no).

#### 2.4.4 Attitude toward death

Death-preparation characteristics comprised thoughts about life-prolonging treatment, medical, ritual, and legal preparation, and perception of a comfortable death. The first component was assessed through responses to the question: “What is your opinion about receiving treatments that prolong life without therapeutic benefit?” Responses were categorized as supportive (strongly or somewhat supportive), unsure, or opposed (somewhat or strongly opposed, or unsure).

Perceptions of a good death were assessed by asking respondents how important they thought the following three sub-contents were during the dying phase. Responses were recorded as either yes or no for each item: “Ritual preparations” (participated in death-preparation education, discussed inheritance/funeral preferences with family, engaged in funeral consultations and mutual aid membership, selected burial site, and prepared shroud or funeral photos); “Legal preparations” (Wrote a will); and “Medical preparations” (Organ or tissue donation).

Perceptions of a good death were assessed by asking respondents how important they considered each of the following aspects at the end-of-life stage: organizing personal affairs before dying, dying without physical or mental pain, being surrounded by family or close acquaintances during death, not burdening them, and dying at home. Respondents rated each item on a 5-point Likert scale ranging from 1 (not important at all) to 5 (very important).

### 2.5 Data analysis

All analyses accounted for the complex-sample design using probability weights. Bivariate associations with AD completion were examined using the Rao-Scott chi-square test (categorical) and survey-adjusted t-test (continuous). Variables significant at *p* < 0.05 in bivariate analyses, together with theoretically relevant sociodemographic factors, were simultaneously input into a survey-weighted multivariable logistic regression model. Because this study was exploratory in nature and was based on nationally representative survey data, variables were selected based on both theoretical relevance and prior literature. Multicollinearity was confirmed to be acceptable (all variance inflation factors [VIFs] < 10; range: 1.01–6.21). Model performance was assessed via goodness-of-fit (F-adjusted Archer-Lemeshow and Hosmer-Lemeshow tests), discrimination (area under the curve [AUC] with 95% CI), and calibration (calibration plot, slope, and Brier score) (Supplementary Figures 1, 2). Analyses were performed using Stata 17.0 (StataCorp, College Station, TX, USA); *p* < 0.05 was considered significant.

## 3 Results

### 3.1 Sociodemographic characteristics and AD completion

The overall completion rate of ADs was 11.1%. AD completion did not differ significantly by gender; however, significant differences were observed based on age and educational attainment. The completion rate was lowest among those aged 65–69 years (9.4%) and highest among those aged 70–74 years (13.2%). A clear educational gradient was also identified, with the lowest completion rate among participants with no formal education (7.7%) and the highest among those with a college education or higher (20.9%). Participants with a religion showed a significantly higher completion rate (14.1%) than those without a religion (9.2%), whereas household size was not significantly associated with AD completion.

Regarding life satisfaction-related variables, satisfaction with health status, social/leisure/cultural activities, social relationships, and overall life satisfaction were significantly associated with AD completion. Participants who completed ADs reported lower satisfaction with their health status. In contrast, their satisfaction with social/leisure/cultural activities, social relationships, and overall life satisfaction was higher than that of non-completers. In addition, the AD-completion rate was highest when medical expenses were paid by the individual or spouse (11.8%) and lower when expenses were covered by children (7.0%) or other payers (6.2%) (Table 1).

**Table 1:** Completion of advance directives by sociodemographic characteristics.

Variable	Category	Advance directive completion			χ^2^/t	*p*
		Total	No	Yes		
		*n*, (%)	*n*, (%)	*n*, (%)a		
		Mean ± SD	Mean ± SD	Mean ± SD		
Total		8,223,404 (100)	1,030,073 (88.9)	9,253,477 (11.1)		
Gender	Male	3,639,342 (44.3)	451,042 (89.0)	4,090,384 (11.0)	0.063	0.801
	Female	4,584,063 (55.7)	579,031 (88.8)	5,163,094 (11.2)		
Age	65–69	2,953,459 (35.9)	307,900 (90.6)	3,261,359 (9.4)	4.984	0.002
	70–74	1,922,995 (23.4)	292,377 (86.8)	2,215,372 (13.2)		
	75–79	1,430,009 (17.4)	181,707 (88.7)	1,611,716 (11.3)		
	80+	1,916,942 (23.3)	248,088 (88.5)	2,165,030 (11.5)		
Education level	No formal education	984,662 (12.0)	81,845 (92.3)	1,066,507 (7.7)	89.482	<0.001
	Elementary school graduate	2,293,778 (27.9)	303,371 (88.3)	2,597,149 (11.7)		
	Middle school graduate	1,800,515 (21.9)	183,894 (90.7)	1,984,409 (9.3)		
	High school graduate	2,623,520 (31.9)	323,160 (89.0)	2,946,681 (11.0)		
	College or higher	520,929 (6.3)	137,802 (79.1)	658,731 (20.9)		
Household size	1–2 persons	7,600,915 (92.4)	967,456 (88.7)	8,568,370 (11.3)	2.979	0.052
	3 persons	481,651 (5.9)	38,183 (92.7)	519,835 (7.4)		
	4 or more persons	140,838 (1.7)	24,434 (85.2)	165,273 (14.8)		
Religion	None	5,031,295 (61.2)	508,186 (90.8)	5,539,481 (9.2)	41.924	<0.001
	Yes	3,192,109 (38.8)	521,887 (86.0)	3,713,997 (14.1)		
Satisfaction	Health status	3.25 ± 0.01	3.26 ± 0.01	3.17 ± 0.03	−2.680	0.007
	Financial status	3.14 ± 0.01	3.13 ± 0.01	3.17 ± 0.03	1.240	0.213
	Social, leisure, and cultural activities	3.15 ± 0.01	3.13 ± 0.01	3.36 ± 0.03	7.760	<0.001
	Social relationships	3.39 ± 0.01	3.38 ± 0.01	3.50 ± 0.03	4.420	<0.001
	overall life satisfaction	3.33 ± 0.01	3.33 ± 0.01	3.41 ± 0.02	3.310	0.001
Burden of medical & caregiving expenses	None	7,585,592 (92.2)	932,527 (89.1)	8,518,119 (11.0)	2.600	0.107
	Yes	637,812 (7.8)	97,546 (86.7)	735,358 (13.3)		
Payer of medical expenses	No medical expenses incurred	407,255 (5.0)	35,697 (91.9)	442,951 (8.1)	5.467	0.001
	Self or spouse	6,966,367 (84.7)	931,447 (88.2)	7,897,814 (11.8)		
	Children	727,390 (8.9)	54,835 (93.0)	782,225 (7.0)		
	Other payers	122,393 (1.5)	8,094 (93.8)	130,487 (6.2)		

### 3.2 Health status and AD completion

Among health-related characteristics, subjective health status itself was not significantly associated with AD completion. However, the number of chronic diseases, number of prescribed medications, depression level, suicidal ideation, and history of falls all showed significant associations with AD completion. The AD-completion rate increased from 8.4% among participants with no chronic disease to 13.8% among those with three or more chronic diseases. A similar pattern was observed for medication use, with completion rates rising from 8.5% among those taking no medication to 14.1% among those taking three or more prescribed medications.

Mental-health indicators were also strongly related to AD completion. The AD-completion rate increased across depression categories, from 10.4% in the normal group to 13.3% in the mild-to-moderate group and 15.4% in the depressed group. Participants with suicidal ideation had a markedly higher AD-completion rate (21.5%) than those without suicidal ideation (11.0%). Likewise, individuals with a history of falls showed a higher AD-completion rate (15.7%) than those without such a history (10.9%) (Table 2).

**Table 2:** Completion of advance directives by health status characteristics.

Variable	Category	Advance directive completion			χ^2^/t	*p*
		Total	No	Yes		
		*n*, (%)	*n*, (%)	n, (%)a		
		Mean ± SD	Mean ± SD	Mean ± SD		
Subjective health status		3.23 ± 0.01	3.23 ± 0.01	3.20 ± 0.03	−0.780	0.437
Number of chronic diseases	0	1,320,621 (14.7)	1,209,181 (91.6)	111,440 (8.4)	10.128	<0.001
	1	2,067,582 (22.9)	1,879,642 (90.9)	187,941 (9.1)		
	2	2,612,436 (28.3)	2,330,461 (89.2)	281,975 (10.8)		
	Three or more	3,252,838 (34.1)	2,804,121 (86.2)	448,717 (13.8)		
Number of prescribed medications	None	1,537,300 (17.1)	1,406,510 (91.5)	130,790 (8.5)	10.184	<0.001
	One	2,222,331 (24.5)	2,010,576 (90.5)	211,754 (9.5)		
	Two	2,681,668 (29.1)	2,390,060 (89.1)	291,609 (10.9)		
	Three or more	2,812,178 (29.4)	2,416,258 (85.9)	395,921 (14.1)		
Depression level	Normal	7,329,324 (79.8)	6,565,818 (89.6)	763,506 (10.4)	7.296	0.001
	Mild to moderate	1,432,861 (15.1)	1,241,831 (86.7)	191,030 (13.3)		
	Depressed	491,292 (5.1)	415,756 (84.6)	75,536 (15.4)		
Suicidal ideation	No	9,158,017 (99.1)	8,148,477 (89.0)	1,009,540 (11.0)	7.662	0.006
	Yes	95,461 (0.9)	74,928 (78.5)	20,533 (21.5)		
History of falls	No	8,774,577 (95.1)	7,819,485 (89.1)	955,092 (10.9)	11.100	0.001
	Yes	478,900 (4.9)	403,920 (84.3)	74,980 (15.7)		

### 3.3 Death-preparation behaviors and AD completion

Most death-preparation variables were strongly associated with AD completion. Participants who opposed life-sustaining treatment exhibited an AD-completion rate of 12.1%, whereas those who were unsure showed the lowest rate (4.0%). Participation in death-preparation education was associated with a substantially higher completion rate (35.7%) compared with non-participation (10.1%). Similarly, AD-completion rates were higher among those who had discussed inheritance or funeral preferences with family (21.4%), received funeral consultation or enrolled in mutual aid programs (19.8%), selected a burial site (17.9%), or prepared a shroud or funeral photograph (13.4%). Overall, individuals who had engaged in practical preparations for death were more likely to complete ADs. Legal- and medical-preparation behaviors showed particularly large differences. The AD-completion rate was 32.2% among those who had written a will and 44.4% among those who had indicated willingness for organ or tissue donation. In addition, participants who completed ADs placed greater importance on components of a good death, including organizing one’s personal affairs, minimizing physical and psychological suffering at the end of life, and not burdening family members (Table 3).

**Table 3:** Completion of advance directives by death-preparation characteristics.

Variable		Category	Advance directive completion			χ2/t	*p*
			Total	No	Yes		
			*n*, (%)	*n*, (%)	*n*, (%)		
			Mean ± SD	Mean ± SD	Mean ± SD		
Thoughts on life-sustaining treatment		Oppose	6,849,937 (83.3)	941,350 (87.9)	7,791,287 (12.1)	16.3247	<0.001
		Unsure	862,439 (10.5)	36,159 (96.0)	898,597 (4.0)		
		Support	511,029 (6.2)	52,564 (90.7)	563,593 (9.3)		
Ritual preparation	Participated in death-preparation education	No	7,976,123 (97.0)	893,029 (89.9)	8,869,152 (10.1)	151.1504	<0.001
		Yes	247,281 (3.0)	137,044 (64.3)	384,325 (35.7)		
	Discussed inheritance/funeral preferences with family	No	7,462,389 (90.8)	823,396 (90.1)	8,285,785 (9.9)	75.6997	<0.001
		Yes	761,016 (9.3)	206,677 (78.6)	967,693 (21.4)		
	Funeral consultations and mutual aid membership	No	6,954,257 (84.6)	716,614 (90.7)	7,670,871 (9.3)	99.5989	<0.001
		Yes	1,269,148 (15.4)	313,459 (80.2)	1,582,606 (19.8)		
	Selected burial site	No	6,643,053 (80.8)	686,398 (90.6)	7,329,451 (9.4)	81.9646	<0.001
		Yes	1,580,351 (19.2)	343,675 (82.1)	1,924,027 (17.9)		
	Prepared shroud or funeral photos	No	5,891,585 (71.6)	668,490 (89.8)	6,560,075 (10.2)	14.6779	0.0001
		Yes	2,331,820 (28.4)	361,583 (86.6)	2,693,402 (13.4)		
Legal preparation	Wrote a will	No	7,935,476 (96.5)	893,564 (89.9)	8,829,040 (10.1)	133.843	<0.001
		Yes	287,929 (3.5)	136,509 (67.8)	424,438 (32.2)		
Medical preparation	Organ or tissue donation	No	7,991,948 (97.2)	845,563 (90.4)	8,837,511 (9.6)	330.1409	<0.001
		Yes	231,456 (2.8)	184,510 (55.6)	415,966 (44.4)		
Perceptions of a good death	Organized personal affairs		4.17 ± 0.01	4.14 ± 0.01	4.36 ± 0.02	8.670	<0.001
	Minimal physical/mental pain at end of life		4.33 ± 0.01	4.31 ± 0.01	4.51 ± 0.03	7.13	<0.001
	Surrounded by family or close acquaintances during death		4.01 ± 0.01	3.99 ± 0.01	4.13 ± 0.03	5.06	<0.001
	Did not burden family or acquaintances		4.24 ± 0.01	4.22 ± 0.01	4.42 ± 0.03	7.16	<0.001
	Dying at home		3.50 ± 0.01	3.49 ± 0.01	3.56 ± 0.03	2.01	0.044

### 3.4 Factors influencing AD completion

Multivariate logistic regression analysis showed that AD completion was independently associated with selected sociodemographic characteristics, depression level, and death-preparation behaviors. Compared with participants aged 65–69 years, those aged 70–74 years were more likely to complete ADs (adjusted odds ratio [aOR] = 1.428, 95% CI, 1.170–1.743, *p* < 0.001). Educational attainment also showed a significant gradient: Compared with those with no formal education, the odds of ADs completion were higher among elementary school graduates (aOR = 1.434, 95% CI, 1.064–1.933, *p* = 0.018) and those with a college education or higher (aOR = 2.428, 95% CI, 1.613–3.655, *p* < 0.001). Having a religion was also positively associated with AD completion (aOR = 1.270, 95% CI, 1.079–1.495, *p* = 0.004).

Among health-related variables, lower health-status scores were associated with lower odds of AD completion (aOR = 0.722, 95% CI, 0.639–0.815, *p* < 0.001). In contrast, greater engagement in social, leisure, and cultural activities (aOR = 1.416, 95% CI, 1.241–1.615, p < 0.001) and better social relationships (aOR = 1.191, 95% CI, 1.023–1.386, *p* = 0.024) were associated with higher odds of AD completion. Depression also remained a significant factor after adjustment, with both the mild-to-moderate (aOR = 1.488, 95% CI, 1.182–1.874, *p* = 0.001) and depressed (aOR = 1.528, 95% CI, 1.058–2.205, p = 0.024) groups showing higher odds of AD completion than the normal group.

Death-preparation behaviors showed the strongest associations with AD completion. Compared with participants who opposed life-sustaining treatment, those who were unsure were significantly less likely to complete ADs (aOR = 0.316, 95% CI, 0.200–0.499, *p* < 0.001), whereas support for life-sustaining treatment was not significantly associated with completion. In contrast, participation in death-preparation education (aOR = 1.539, 95% CI, 1.032–2.294, *p* = 0.034), funeral consultation or mutual aid membership (aOR = 1.566, 95% CI, 1.260–1.945, *p* < 0.001), burial-site selection (aOR = 1.453, 95% CI, 1.191–1.773, p < 0.001), writing a will (aOR = 1.894, 95% CI, 1.277–2.810, *p* = 0.002), and willingness to donate organs or tissue (aOR = 4.473, 95% CI, 3.167–6.319, *p* < 0.001) were all significantly associated with increased odds of AD completion. Among perceptions of a good death, only valuing the organization of one’s personal affairs remained independently significant (aOR = 1.411, 95% CI, 1.233–1.616, *p* < 0.001) (Table 4; Supplementary Figures 1, 2).

**Table 4:** Factors influencing completion of advance directives.

Variable	Category		aOR	*p*	95% CI
Gender	Male		1		
	Female		1.050	0.575	0.885–1.247
Age	65–69		1		
	70–74***		1.428	0.000	1.170–1.743
	75–79		1.171	0.207	0.916–1.497
	80+		1.226	0.153	0.927–1.620
Education level	No formal education		1		
	Elementary school graduate*		1.434	0.018	1.064–1.933
	Middle school graduate		1.145	0.439	0.813–1.613
	High school graduate		1.386	0.064	0.981–1.958
	College or higher***		2.428	0.000	1.613–3.655
Religion	None		1		
	Yes**		1.270	0.004	1.079–1.495
Health status***			0.722	0.000	0.639–0.815
Social, leisure, and cultural activities***			1.416	0.000	1.241–1.615
Social relationships*			1.191	0.024	1.023–1.386
overall life satisfaction			1.095	0.325	0.914–1.313
Burden of medical & caregiving expenses	None		1		
	Yes		0.997	0.987	0.731– 1.361
Payer of medical expenses	No medical expenses incurred		1		
	Self or spouse		1.559	0.058	0.985–2.468
	Children		0.862	0.617	0.481–1.543
	Other payers		0.360	0.186	0.079–1.634
Number of chronic diseases	0		1		
	1		1.086	0.798	0.577–2.044
	2		1.279	0.438	0.687–2.384
	Three or more		1.520	0.219	0.779–2.967
Number of prescribed medications	None		1		
	One		0.997	0.993	0.553–1.797
	Two		0.898	0.716	0.501–1.607
	Three or more		0.923	0.802	0.493–1.728
Depression level	Normal		1		
	Mild to moderate**		1.488	0.001	1.182–1.874
	Depressed*		1.528	0.024	1.058–2.205
Suicidal ideation	No		1		
	Yes		1.866	0.069	0.953–3.656
History of falls	No		1		
	Yes		1.258	0.131	0.934–1.694
Thoughts on life-sustaining treatment	Oppose		1		
	Unsure***		0.316	0.000	0.200–0.499
	Support		0.927	0.673	0.651–1.319
Ritual preparation	Participated in death-preparation education*	None	1		
		Yes	1.539	0.034	1.032–2.294
	Discussed inheritance/funeral preferences with family	None	1		
		Yes	1.258	0.107	0.951–1.663
	Funeral consultations and mutual aid membership***	None	1		
		Yes	1.566	0.000	1.260–1.945
	Selected burial site***	None	1		
		Yes	1.453	0.000	1.191–1.773
	Prepared shroud or funeral photos	None	1		
		Yes	0.928	0.464	0.759–1.134
Legal preparation	Wrote a will**		1.894	0.002	1.277–2.810
Medical preparation	Organ or tissue donation***		4.473	0.000	3.167–6.319
Perceptions of a good death	Organized personal affairs***		1.411	0.000	1.233–1.616
	Minimal physical/mental pain at end of life		1.132	0.090	0.981–1.307
	Surrounded by family or close acquaintances during death		0.971	0.601	0.869–1.085
	Did not burden family or acquaintances		1.122	0.099	0.979–1.287
	Dying at home		0.942	0.179	0.863–1.028

aOR = adjusted odds ratio; CI = confidence interval; AUC = area under the curve. Reference categories are indicated in parentheses. Model diagnostics: F-adjusted goodness-of-fit test, *F* (9, 9,787) = 1.08, *p* = 0.376; Hosmer-Lemeshow χ^2^ (8) = 8.58, *p* = 0.379; AUC = 0.760 (95% CI: 0.744–0.775); calibration slope = 1.000; Brier score = 0.087. *p < 0.05, **p < 0.01, ***p < 0.001.

## 4 Discussion

This study analyzed the determinants of AD completion among Korean older adults using a representative sample from the 2023 National Survey of Older Koreans. The results indicate that AD completion is linked not only to sociodemographic factors but also to health perception, mental-health status, and comprehensive death-preparedness behaviors (16, 19, 22).

First, a paradoxical relationship exists between subjective health status and AD completion. In this study, the finding that older adults with better subjective health were less likely to complete ADs can be interpreted as an “optimistic bias” during periods of good health (13, 17). This aligns with the tendency of older adults to view death as a distant event when they positively perceive their health, thereby postponing end-of-life planning (1, 13, 24). This also relates to attitude formation within the TPB (13). Conversely, older adults experiencing chronic diseases or polypharmacy more acutely perceive the trajectory of illness, leading to a stronger recognition of the necessity for ADs (20, 21). Therefore, differentiated education strategies are required to engage healthy older adults in the ACP process (15, 25).

Second, the positive association between mental-health status (specifically depression and suicidal ideation) and AD completion may reflect important psychological dimensions related to end-of-life decision-making. Depressive symptoms and suicidal ideation, assessed via validated instruments such as the Short Form of the Geriatric Depression Scale-Korean version (SGDS-K), displayed a significant association with AD completion (2, 23, 26). The higher rate of AD completion observed among older adults experiencing psychological distress suggests that they may utilize ACP as a means of securing a sense of psychological control and autonomy over end-of-life decisions (26, 27). Previous studies have similarly suggested that existential and psychological suffering may influence attitudes toward dignified death and end-of-life preparation (28). Consequently, for these psychologically vulnerable older adults, professional counseling and psychosocial support should be integrated into the AD-completion process to help ensure psychologically supported and well-informed decision-making (18, 24).

Third, the “comprehensive connectivity” of death-preparedness behaviors was strongly associated with AD completion. Practical preparations, such as organ-donation intention (aOR 4.47), writing a will, and funeral consultations, were the strongest predictors of AD completion (29, 30). These observations suggest that for Korean older adults, completing ADs is perceived not as a standalone medical procedure but as part of a comprehensive “rite of passage” to organize one’s life (31, 32). This approach aligns with recent global public-health paradigms shifting toward a more dynamic, communication-centered framework for death preparedness (32). In particular, the strong link between organ-donation intention—reflecting altruistic values—and AD completion demonstrates the spread of end-of-life awareness grounded in ego integrity and spiritual well-being (33, 34), a multi-dimensional correlation consistently validated by comprehensive international systematic reviews of ACP behaviors (34).

Last, the clarity of knowledge regarding life-sustaining treatment and the role of education was confirmed. The significantly lower completion rate among those who responded “do not know” to life-sustaining treatment indicates that information asymmetry is a practical barrier to ACP participation (35, 36). The significant increase in completion rates associated with participation in death education supports the effectiveness of standardized public education (37, 38). To bridge gaps related to education levels and social support, equitable policy considerations for information-poor older adults must be continually strengthened, a priority underscored by recent global meta-analytic evidence advocating structured intervention frameworks for vulnerable populations (39, 40).

By identifying the multidimensional determinants of AD completion using the latest data from 9,796 individuals, this study provides empirical evidence that may inform discussions on end-of-life preparation and ACP-related support systems in an aging society, which holds significant academic value (12, 25).

## 5 Conclusion

In conclusion, this study identifies key behavioral and social correlates of AD completion among older Korean adults. Rather than justify immediate institutional mandates, these findings provide essential empirical baseline data to optimize community-guided educational frameworks and support autonomous end-of-life care planning.

## 6 Implications and limitations

### 6.1 Implications

This study’s findings provide several critical insights for policy development and clinical practice aimed at enhancing ACP among the older population. First, a definite need exists for target-specific educational strategies with a dual-track approach. For relatively healthy seniors who may exhibit an optimistic bias toward their longevity, the focus should be on preventive education that emphasizes the proactive right to a dignified death and personal autonomy. Conversely, for seniors managing chronic illnesses, a procedural strategy is required to provide concrete information on medical interventions and life-sustaining treatment options that reflect their specific clinical realities. Furthermore, the significant correlation between psychological distress and the completion of ADs necessitates the integration of registration processes with professional mental-health support systems. Rather than view these legal documents as a byproduct of despair, systems should provide specialized counseling that helps distressed seniors reframe AD completion as a constructive act of healthy life closure and self-determination. Finally, policies should move toward a more holistic framework of death preparedness by linking AD registration with other preparatory actions, such as organ donation and estate planning, to normalize the process within the broader context of well-dying. Consequently, public-health strategies should focus on integrating community-level educational interventions with existing welfare networks, rather than pursue top-down administrative mandates, to better support older adults’ autonomy.

### 6.2 Limitations

Despite these contributions, this study has several limitations that warrant consideration in future research. Primarily, because this study used data from the 2023 National Survey of Older Koreans, its cross-sectional design makes it difficult to establish definitive causal relationships between the identified variables and the completion of ADs. Future longitudinal studies are necessary to track how evolving health or psychological statuses over time influence long-term planning behaviors. Additionally, reliance on self-reported survey responses introduces the potential for social-desirability bias, in which participants might over-report their completion of ADs to align with perceived social norms. Future research should aim to validate these findings by triangulating survey data with official government records from the National Agency for Management of Life-Sustaining Treatment. Finally, while the analysis was based on a robust sample of 9,796 individuals, the study was limited to community-dwelling older adults and excluded those residing in long-term care facilities or those with severe cognitive impairments such as advanced dementia. Therefore, caution is warranted when generalizing these results to the entire older population, and subsequent studies should employ more inclusive sampling methods that include these vulnerable groups.

## Data Availability

Publicly available datasets were analyzed in this study. This data can be found at Health and Welfare Data Portal (Korea Institute for Health and Social Affairs), Repository URL: https://data.kihasa.re.kr, Statistical Approval No. 117071 (KOSTAT).
