# Supplementary material for: Determinants of advance-directive completion for end-of-life preparedness among older Korean adults
Source: Front Public Health. 2026 Jun 30;14:1839072. doi: 10.3389/fpubh.2026.1839072 (PMC13366214; doi:10.3389/fpubh.2026.1839072)
Supplement: Supplementary file 1 [file Table_1.DOCX]

Supplementary Material

# Supplementary Figures

**Supplementary Figure 1.** ROC Curve. Receiver operating characteristic (ROC) curve for the final multivariable logistic regression model predicting advance directive completion. AUC = 0.760 (95% CI: 0.744–0.775)

**Supplementary Figure 2.** Calibration Plot. Calibration plot comparing observed and predicted probabilities of advance directive completion across deciles of predicted risk. The dashed red line represents ideal calibration (y = x). Calibration slope = 1.000; Brier score = 0.087.
